# A direct stimulatory role of mobile gibberellin in *Arabidopsis* hypocotyl xylem expansion

**DOI:** 10.1186/1753-6561-5-S7-P73

**Published:** 2011-09-13

**Authors:** Kaisa Nieminen, Laura Ragni, David Pacheco-Villalobos, Richard Sibout, Christian S Hardtke

**Affiliations:** 1 Department of Plant Molecular Biology, University of Lausanne, Switzerland

## Background

Can Arabidopsis research contribute to our understanding about wood development? Does the function of vascular cambium in a herbaceous weed resemble that of a tree? Despite its diminutive size as compared to a tree, Arabidopsis still displays cambial driven secondary thickening in several organs, including the hypocotyl. Hypocotyl is a good model organ for wood development studies, as in this organ the radial secondary development can be uncoupled from the apical primary growth. This is due to the fact that the hypocotyl elongates only for five days after germination; thus, the radial secondary growth starts only after the elongation has ceased. This is in contrast to the other Arabidopsis organs displaying cambial growth, where it is accompanied by the simultaneous activity of the shoot and root meristems.

**Figure 1:**
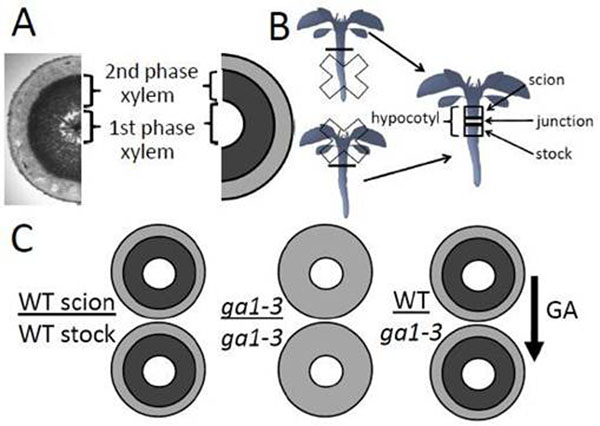
A) Cross-section of Arabidopsis hypocotyl showing the two phases of secondary development. B) In Arabidopsis grafting, young seedlings are cut into two through hypocotyl, and the hypocotyl of shoot (scion) part and the root (stock) part are then re-attached in the desired combination. C) Schematic picture of the *ga1-3* vs wild-type (WT) grafting experiment. GA from the WT scion was able to rescue xylem expansion in the hypocotyl of the GA deficient *ga1-3* stock.

Two phases can be identified in the hypocotyl secondary development: 1) an early phase of proportional radial growth, where the cambium produces both xylem and phloem at a similar rate, and 2) a later xylem expansion phase, where more xylem than phloem is produced (Fig 1A) [1] . Notably, the composition of xylem is different between these two phases: the xylem produced during the first phase consists of xylem vessels and parenchyma cells, and of xylem vessels and fibers during the second phase. Especially the later phase, characterized by extensive wood formation, resembles the secondary growth in tree species.

We have previously shown that in Arabidopsis hypocotyl the transition from the first to the second phase is triggered through the onset of flowering, when the identity of shoot apical meristem changes from vegetative to reproductive [1] . Upon this transition, instead of new leaves, an inflorescence stem emerges from the middle of rosette leaves. What could be the nature of this signal [2]?

## Results and discussion

Does flower differentiation trigger the onset of xylem expansion? To study this, we analyzed Arabidopsis null mutants for key flowering regulators. We were able to confirm that xylem expansion took place in all our mutants, suggesting that neither floral specification nor bolting (the emergence and elongation of the inflorescence stem from the rosette) are required for this process.

Thus, our signal appeared to be upstream of flowering; possibly the same signal activated both flowering and xylem expansion? Since gibberellin hormone (GA) has been shown to be an important regulator of flowering initiation, we wondered what effect it would have on the secondary growth. Indeed, both xylem expansion and flowering were initiated upon GA treatment. To study if GA biosynthesis was required for xylem expansion, we analyzed *ga1-3*, a null mutant for the *GA REQUIRING 1* gene encoding a key GA biosynthesis enzyme. As expected, these plants displayed strongly reduced xylem expansion.

Next we wanted to study, if not only the GA content, but the actual level of GA signaling, was important for the regulation of xylem expansion. We observed that the transgenic and mutant lines with elevated GA signaling displayed increased and the lines with impaired GA signaling accordingly reduced xylem expansion, thus confirming the rate-limiting role of GA in this process.

Does GA signaling stimulate xylem expansion at the cambium, or does it function at the shoot apex to launch the production of the mobile signal? To study this, we performed grafting experiments (Fig 1B). We could see that the wild-type level of GA signaling in either part of the graft could not rescue the xylem expansion if the other part was dominantly inhibited in GA signaling. Accordingly, elevated GA signaling level enhanced xylem expansion only locally; the effect was not graft-transmissible to the wild-type part of the graft. Thus, GA signaling cascade appears to act as a local regulator of cambial activity, downstream of the mobile signal.

Could the GA hormone itself be mobile? We studied this by reciprocally grafting the GA deficient *ga1-3* mutant with a wild-type plant. Strikingly, in these plants, the wild-type scion (shoot part) restored xylem expansion in the hypocotyl of the *ga1-3* stock (root part) (Fig 1C). To sum up, an impaired GA signaling did not affect xylem expansion systemically, suggesting that it acts downstream of the mobile cue. By contrast, the GA effect was graft transmissible, confirming that GA itself is the mobile shoot-derived signal.

## Conclusions

Our study shows that GA acts as a mobile signal that activates the onset of extensive xylem production in Arabidopsis hypocotyl. It would be interesting to study if a similar GA driven process takes place also in tree species upon the seasonal or age-related activation of their cambial growth. We think that Arabidopsis research has the potential to contribute novel hypotheses into secondary development research, and that it can complement tree studies in some areas of this research field. However, due to the sheer extend of secondary development present in tree species, and their other special characteristics (the seasonal activity-dormancy cycle etc.), many processes of secondary growth remain to be best studied in trees.
